# Supplementary material for: The pesticide chlorpyrifos promotes obesity by inhibiting diet-induced thermogenesis in brown adipose tissue
Source: Nat Commun. 2021 Aug 27;12:5163. doi: 10.1038/s41467-021-25384-y (PMC8397754; doi:10.1038/s41467-021-25384-y)
Supplement: Supplementary file 1 — Supplementary Information [file 41467_2021_25384_MOESM1_ESM.pdf]

SUPPLEMENTARY INFORMATION

Supplementary Table 1: List of genes and respective Taqman Assay IDs used for RT-qPCR gene expression analysis that were used in multiple figures.

Gene Name	Assay ID
Ucp1	Mm01244861_m1
Cox8b	m00432648_m1
Ppargc1a	Mm00447183_m1
Ppia	Mm02342430_g1
Prdm16	Mm00712556_m1
Pparg	Mm00440940_m1
Adrb3	Mm02601819_g1
Adcy3	Mm00460371_m1
Zfp516	Mm00813584_m1

Supplementary Figures 1-10

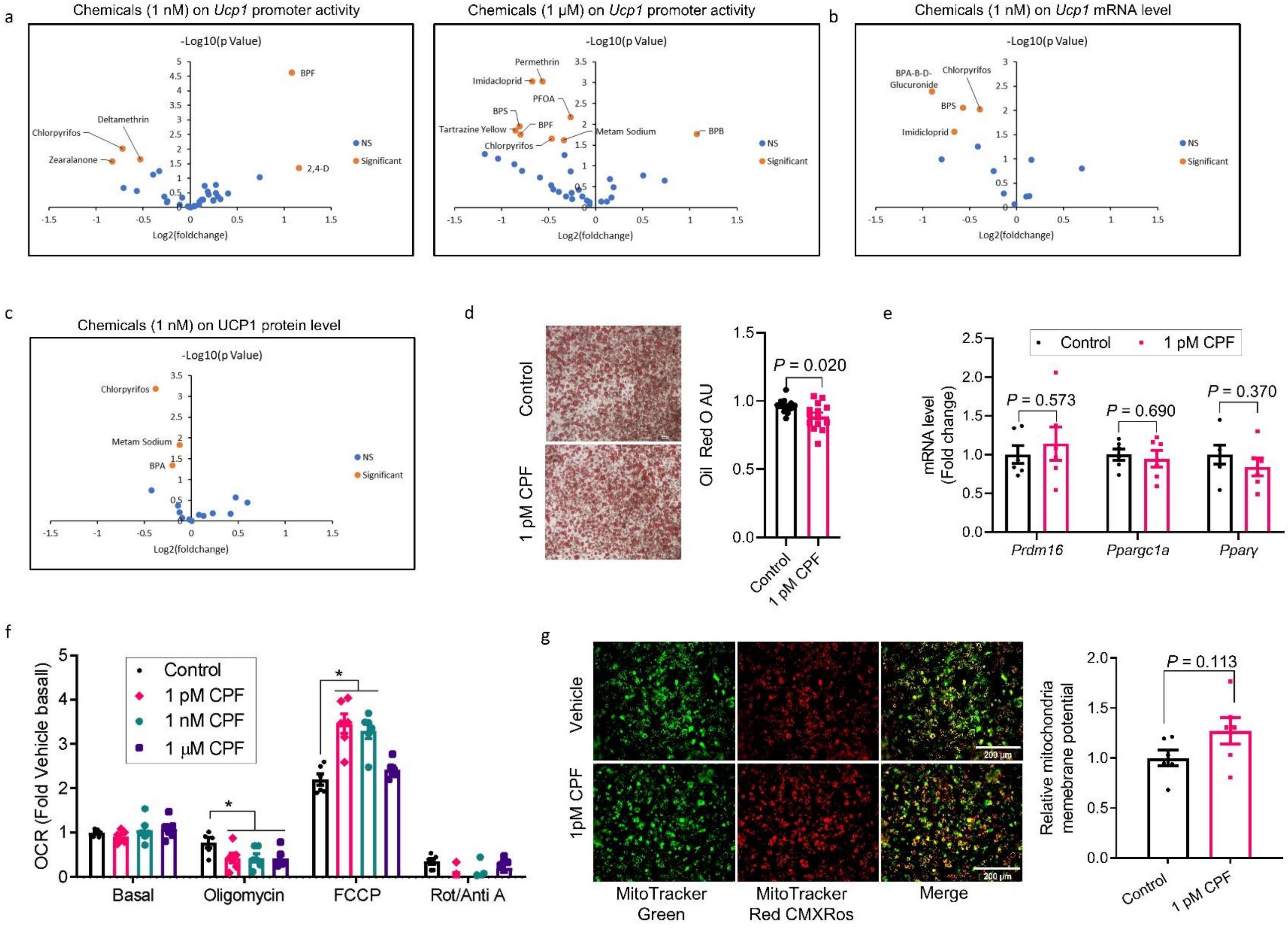

Supplementary Figure 1. CPF inhibits UCP1 and mitochondrial respiration in cultured brown adipocytes. a, Change in *Ucp1* promoter activity. **b**, *Ucp1* mRNA level and **c**, UCP1 protein level in brown adipocytes treated with 34 different chemicals at the dose of 1 nM and 1 μM. **d-e**, Oil red O staining (d) and brown adipogenic genes (e) of mature brown adipocytes treated with 1 pM chlorpyrifos (CPF) for 7 days. **f**, Oxygen consumption rate of brown adipocytes treated with different doses of CPF for 4 hours. **g**, Representative images of mitochondria stained by MitoTracker Green and quantification of mitochondria potential reflected by MitoTracker Red in brown adipocytes treated with 1 pM CPF for 1 hour. Significant differences between 3 or more mean values were determined by one-way ANOVA with Tukey's multiple comparisons test; differences between 2 mean values were determined by Student's t test. Significant differences between 3 or more mean values were determined by one-way ANOVA with the post hoc Bonferroni's multiple comparisons test; differences between 2 mean values were determined by two-tailed Student's t-test. Data presented are mean ± SEM, n = 6, * $p < 0.05$. Scale bar = 200 μm.

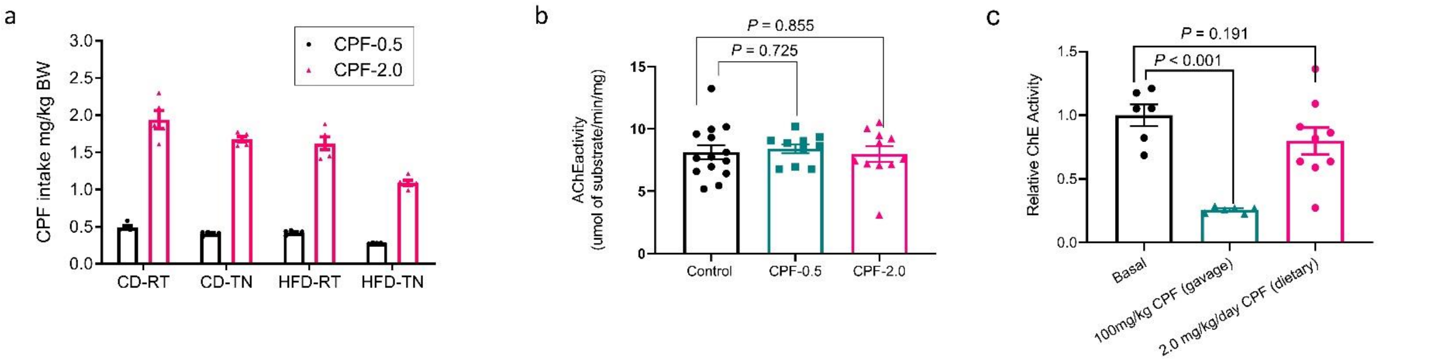

Supplementary Figure 2. CPF intake and the Effects of CPF on AChE activity. a, Chlorpyrifos (CPF) intake of C57BL/6J male mice fed with CD (10 kcal% fat) or HFD (45 kcal% fat) supplemented with 0 (Control), 0.5 mg/kg/BW (CPF-0.5) or 2.0 mg/kg/BW (CPF-2.0) at room temperature (RT, 22 °C) or thermoneutrality (TN, 30 °C), n = 5. **b**, AChE activity assessed in hindlimb skeletal muscle of CPF treated mice, n = 12. **c,** ChE activity in serum of C57BL/6J mice at 4h post gavage of 100 mg/kg CPF (n = 6) or after treatment with 2.0 mg/kg/day CPF for 3 weeks (n = 10), relative to basal. Significant differences between 3 or more mean values were determined by one-way ANOVA with the post hoc Bonferroni's multiple comparisons test; differences between 2 mean values were determined by two-tailed Student's *t*-test. Data presented are mean ± SEM.

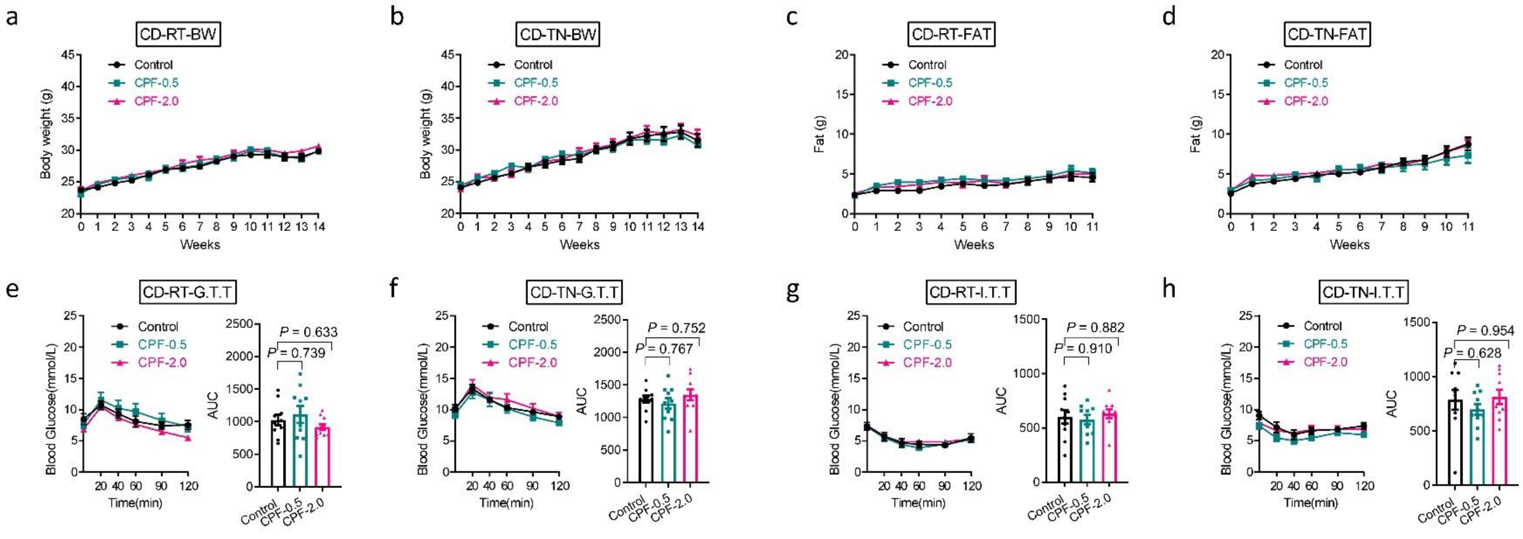

Supplementary Figure 3. Effects of CPF on body weight, fat mass and glucose metabolism of mice fed a control diet.

C57BL/6J male mice were treated with CD (10 kcal% fat) supplemented with 0 (Control), 0.5 mg/kg/BW (CPF-0.5) or 2.0 mg/kg/BW (CPF-2.0) at room temperature (RT, 22 °C) or thermoneutrality (TN, 30 °C). **a-b**, Body weight of mice at RT (**a**) or TN (**b**). **c-d**, Fat mass of mice at RT (**c**) or TN (**d**). **e-f**, Glucose tolerance test (GTT) of mice at RT (**e**) or TN (**f**). **g-h**, Insulin tolerance test (ITT) of mice at RT (**g**) or TN (**h**). Significant differences between mean values were determined by one-way ANOVA with Tukey's multiple comparisons test. Significant differences between mean values were determined by one-way ANOVA with the post hoc Bonferroni's multiple comparisons test. Data presented are mean ± SEM, n = 10.

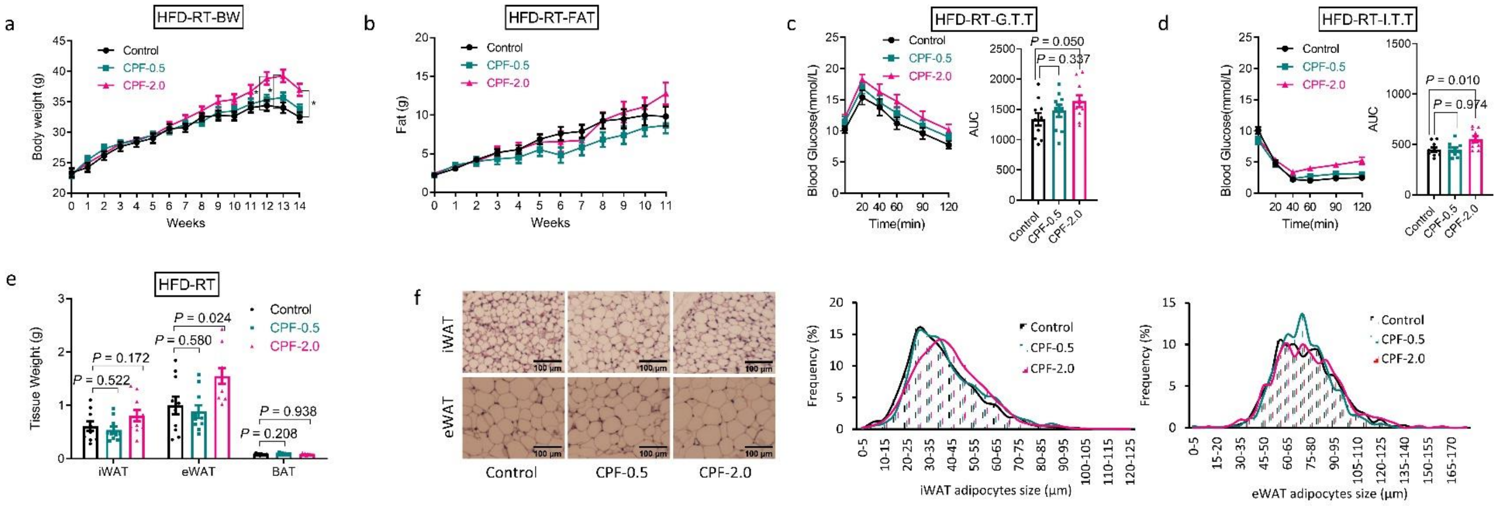

Supplementary Figure 4. Effects of CPF on body weight and glucose metabolism of mice fed a high-fat diet at room temperature.

C57BL/6J male mice were treated with HFD (45 kcal% fat) supplemented with 0 (Control), 0.5 mg/kg/BW (CPF-0.5) or 2.0 mg/kg/BW (CPF-2.0) at room temperature (RT, 22 °C). **a**, Body weight (BW). **b**, Fat mass. **c**, Glucose tolerance test (GTT). **d**, Insulin tolerance test (ITT). **e**, Fat tissues weight. **f**, Representative images of inguinal white adipose tissue (iWAT) and epididymal white adipose tissue (eWAT) adipocytes and the quantification of adipocyte size. Significant differences between mean values were determined by one-way ANOVA with the post hoc Bonferroni's multiple comparisons test. Data presented are mean ± SEM, n = 10, * $p < 0.05$. Scale bar = 100 μm.

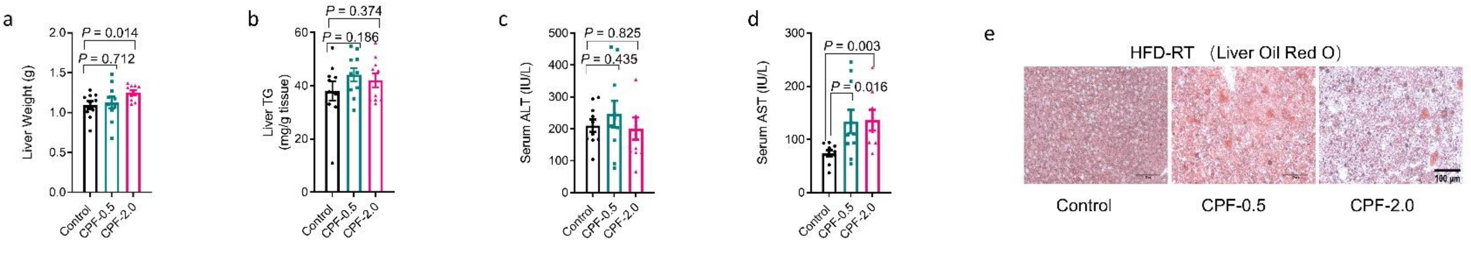

Supplementary Figure 5. Effects of CPF on NAFLD in mice fed a high-fat diet at room temperature.

C57BL/6J male mice were treated with HFD (45 kcal% fat) supplemented with 0 (Control), 0.5 mg/kg/BW (CPF-0.5) or 2.0 mg/kg/BW (CPF-2.0) at room temperature (RT, 22 °C). **a**, Liver weight. **b**, Liver TG. **c**, Serum alanine aminotransferase (ALT) level. **d**, aspartate aminotransferase (AST) level. **e**, Representative images of Oil Red O stained liver lipids. Significant differences between mean values were determined by one-way ANOVA with the post hoc Bonferroni's multiple comparisons test. Data presented are mean ± SEM, n = 10. Scale bar = 100 μm.

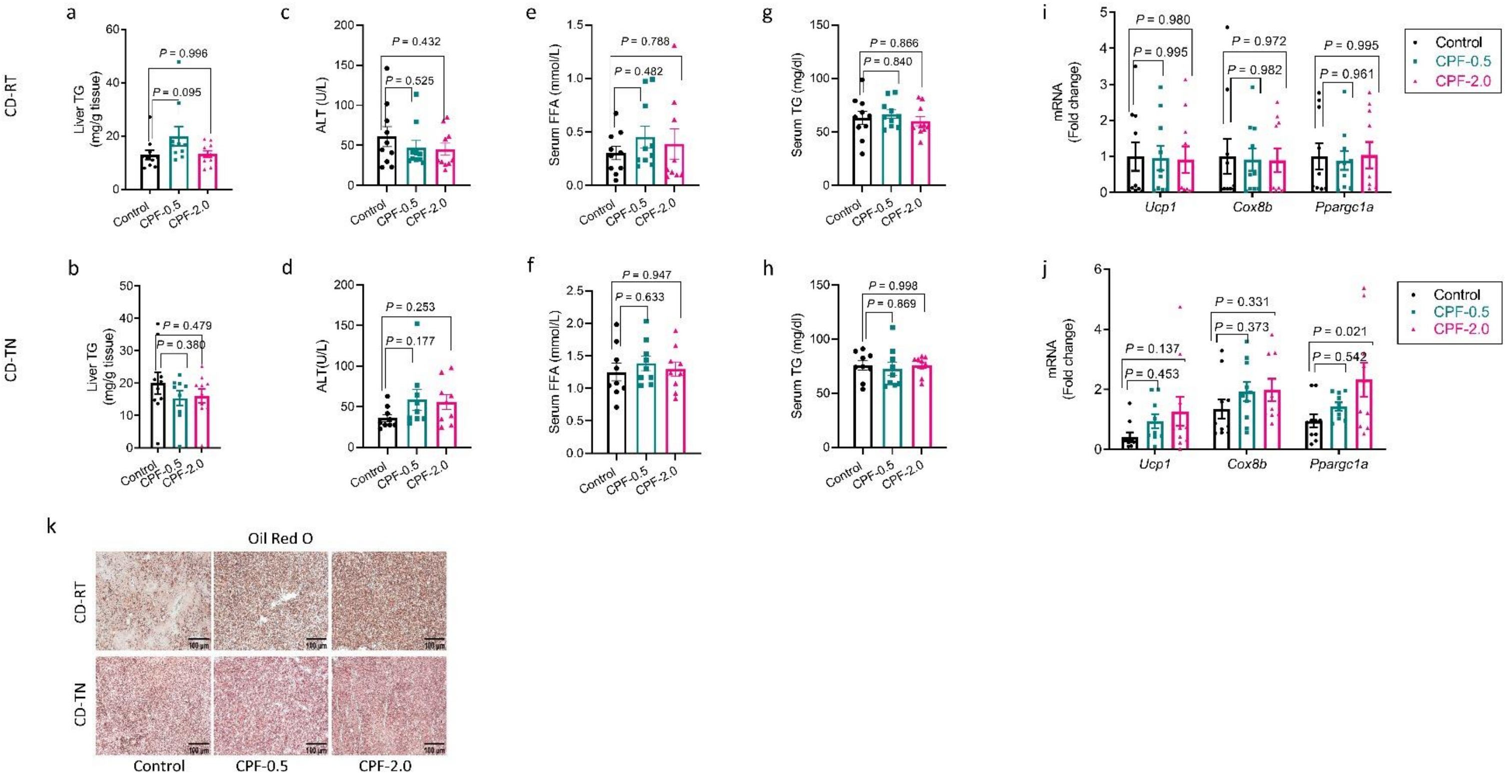

Supplementary Figure 6. Effects of CPF on NAFLD in mice fed a control diet.

C57BL/6J male mice were treated with CD (10 kcal% fat) supplemented with 0 (Control), 0.5 mg/kg/BW (CPF-0.5) or 2.0 mg/kg/BW (CPF-2.0) at room temperature (RT, 22 °C) or thermoneutrality (TN, 30 °C). **a-b**, Liver TG of mice at RT (a) or TN (b). **c-d**, Serum alanine aminotransferase (ALT) level of mice at RT (c) or TN (d). **e-f**, serum free fatty acid (FFA) of mice at RT (e) or TN (f). **g-h**, serum triacylglycerol (TG) of mice at RT (g) or TN (h). **i-j**, mRNA level of brown adipose genes in BAT of mice at RT (i) or TN (j). **k**, Representative images of Oil Red O stained liver lipids Significant differences between mean values were determined by one-way ANOVA with the post hoc Bonferroni's multiple comparisons test. Data presented are mean ± SEM, n = 10. Scale bar = 100 μm.

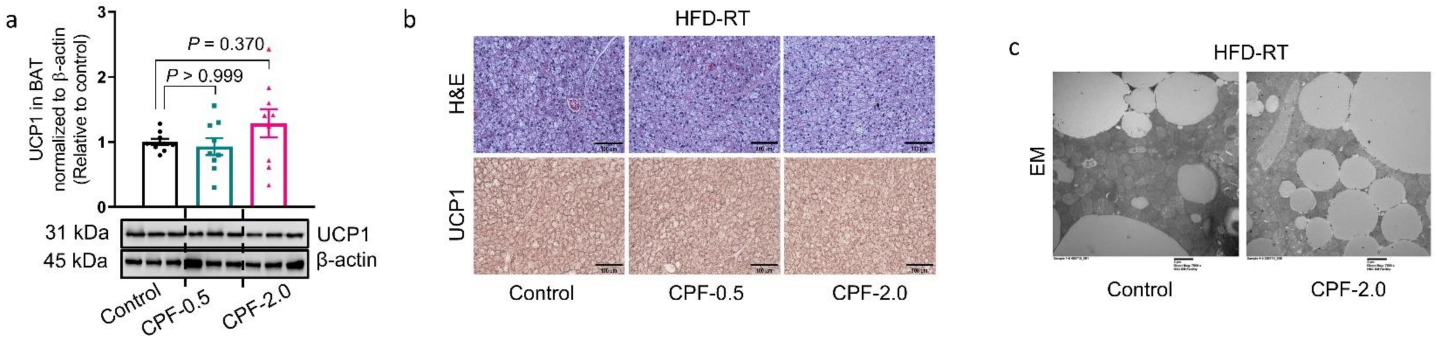

Supplementary Figure 7. Effects of CPF on BAT of mice fed a HFD at room temperature.

C57BL/6J male mice were treated with HFD (45 kcal% fat) supplemented with two doses of CPF at room temperature (RT). **a**, UCP1 protein content in BAT. **b**, Representative immunohistochemistry images showing UCP1 in BAT and H&E images, Scale bar = 100 μm. **c**, Representative electron micrographs for mitochondria, scale bar = 2 μm. Significant differences between mean values were determined by one-way ANOVA with the post hoc Bonferroni's multiple comparisons test. Data presented are mean ± SEM, n = 10.

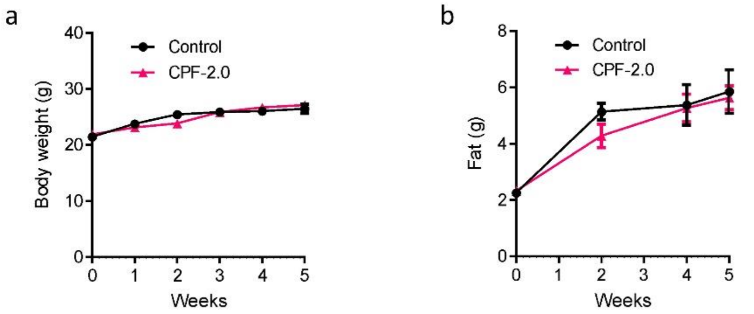

Supplementary Figure 8. 5-week CPF exposure does not alter body weight or fat content. a-b, body weight (a) and fat content (b) of C57BL/6J male mice fed with a HFD (45 kcal% fat) supplemented with 0 (control) or 2.0 mg/kg/BW (CPF-2.0) chlorpyrifos for 5 weeks. n = 10. Significant differences between mean values were determined by two-tailed Student's *t*-test, no differences between means were detected. Data presented are mean ± SEM.

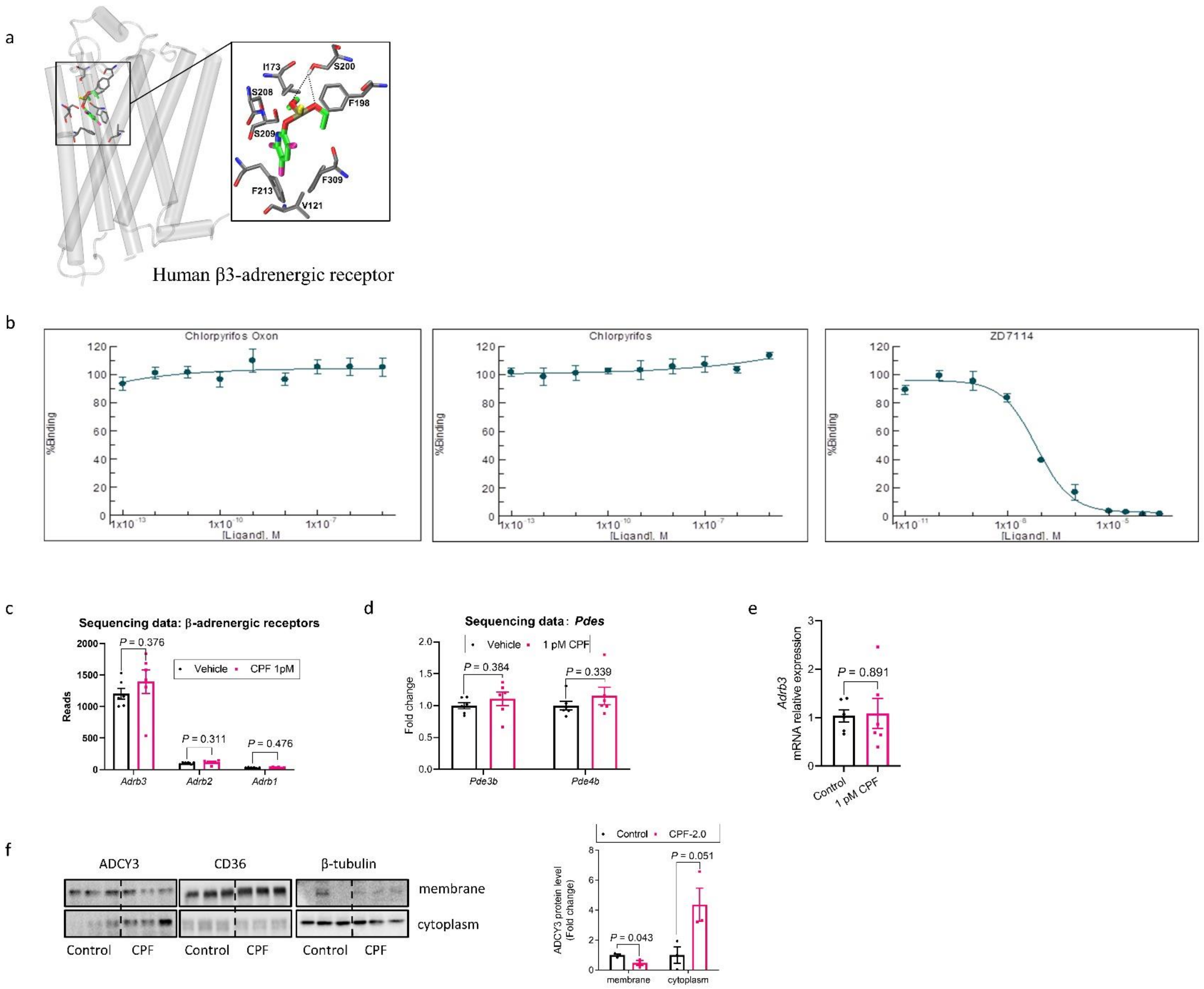

Supplementary Figure 9. Chlorpyrifos (CPF) alters cytoplasmic ADCY3 levels and does not competitively bind to b3-AR. a, Docked pose of chlorpyrifos (green stick) in human β3-adrenergic receptor represented using gray cartoon. Amino acid residues (gray stick) highlighting the predicted binding site of chlorpyrifos are shown in an enlarged view. Nitrogen, Oxygen, Sulfur, Phosphorus, Chlorine and Hydrogen atoms are shown using blue, red, yellow, bronze, pink and white colors respectively. Black dashed line indicates hydrogen bond interaction. **b**, Radioligand competition binding curves of CPF and CPF-oxon binding on the human recombinant ADRB3, ZD7114 was utilized as a positive control, n = 3. **c-d** mRNA sequencing data of *Adrbs* and *Pdes* in mature brown adipocytes treated with 1 pM CPF for 4 h (n = 6). **e,** *Adrb3* mRNA expression in mature brown adipocytes treated with 1 pM CPF for 7d (n = 6). **f,** Subcellular localization of ADCY3 (g) in BAT of C57BL/6J male mice treated with HFD (45 kcal% fat) supplemented with 0 (control) or 2.0 mg/kg/BW (CPF-2.0) CPF at thermoneutral condition (TN, 30 °C, n = 3). Significant differences between mean values were determined by two-tailed Student's *t*-test. Data presented are mean ± SEM.

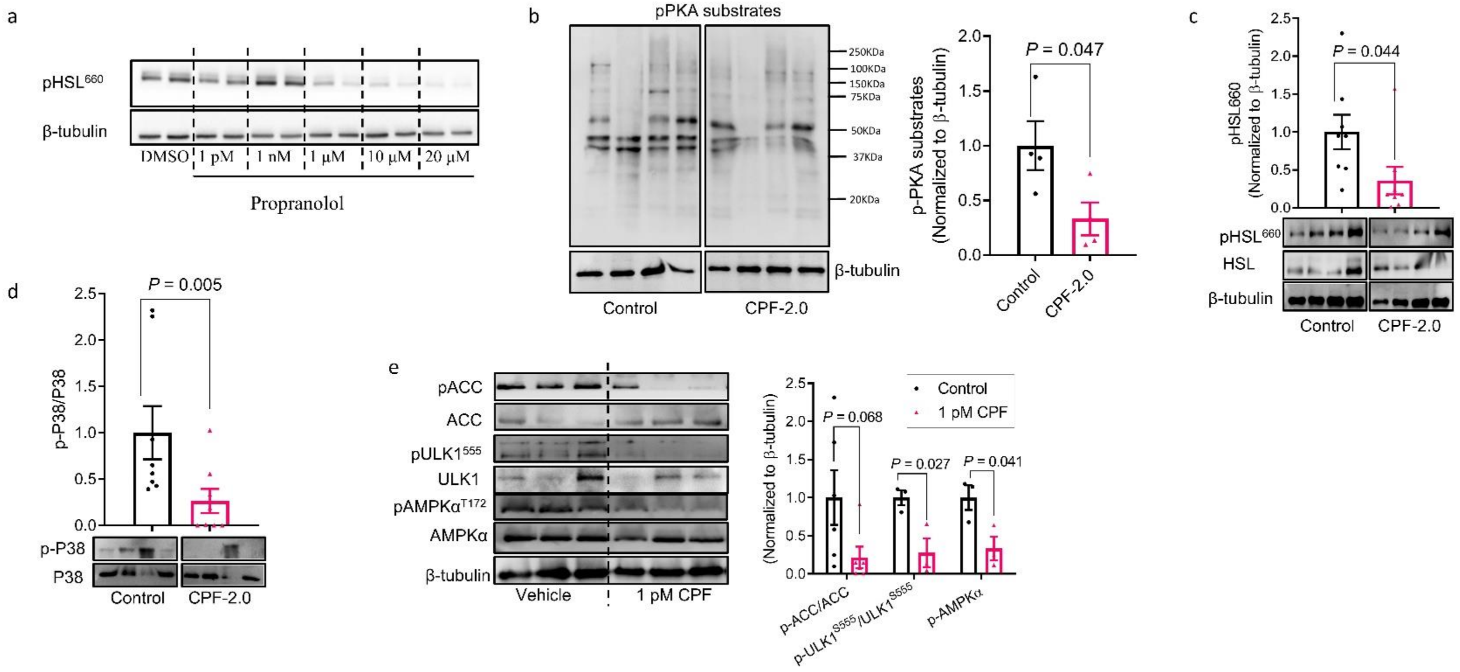

Supplementary Figure 10. Inhibitory role of CPF on downstream targets of BAT thermogenesis a. Mature brown adipocytes treated with propanol for 72 h followed by a 3 min isoproterenol treatment at 10 nM, phosphorylation of HSL was analyzed. **b-d**, pPKA, pHSL660, HSL, p-P38 and P38 protein in BAT of of C57BL/6J mice fed a HFD with 0 (Control) or 2.0 mg/kg/BW (CPF-2.0) chlorpyrifos (CPF) and housed at thermoneutrality (TN, 30 °C) for 14weeks (n = 8). **e**. pACC, ACC, pULK1^{555}, ULK1, pAMPKα ^{Thr172} and AMPKα in mature brown adipocytes treated with 1 pM CPF for 7 days (n = 6). Significant differences between mean values were determined by two-tailed Student's *t*-test. Data presented are mean ± SEM.
